# 555. Clinical impact and real-life utilization of Karius testing: a retrospective analysis of cases from an urban, academic medical center

**DOI:** 10.1093/ofid/ofad500.624

**Published:** 2023-11-27

**Authors:** Liam Conway-Pearson, Erika Orner, Phyu Thwe, Wendy Szymczak, Margaret E McCort

**Affiliations:** Montefiore Medical Center, New York, New York; Montefiore Medical Center, New York, New York; Montefiore Medical Center, New York, New York; Montefiore Medical Center, Albert Einstein College of Medicine, Bronx, NY; Montefiore Medical Center / Albert Einstein College of Medicine, Bronx, New York

## Abstract

**Background:**

Metagenomic next-generation sequencing (NGS) of microbial cell-free DNA is a relatively novel diagnostic technique that may potentially expedite and/or supplement pathogen-specific infectious disease diagnoses, minimize the time to appropriate antibiotic therapy, and reduce unnecessary and invasive testing. However, careful diagnostic stewardship is necessary to ensure that such tests are being ordered in the appropriate patient population.

**Methods:**

A retrospective chart review of all patients for whom the Karius NGS test was sent at our institution between Jun 13, 2020-Jul 22, 2022 was performed. Clinically relevant pathogens were defined as diagnoses that led to change in antimicrobial therapy. Stata statistical software (version 17.0, College Station TX) was used to analyze patient and hospitalization risk factors associated with Karius testing.

**Results:**

Thirty cases among 29 unique patients for whom Karius testing was performed were included in our review. Patient characteristics are shown in Table 1. Twenty-one patients (72%) were immunocompromised, including 14 (47%) with solid organ transplant (SOT) and 9 (30%) with malignancy. Mean hospital length of stay (LOS) prior to Karius test collection was 15.8 days (+/- 21.6 days, range 1-120). Reasons for sending Karius are shown in Fig 1. Three patients (10%) had tissue-specific NGS testing performed in addition to Karius. On average, patients received 13.9 days of antibiotics prior to Karius (+/-10.11, range 0-37). Mean turnaround time for Karius results was 38.3 hours (+/- 16.5, range 23-79). Ten (34.5%) Karius results identified no pathogens. Karius identified fungal organisms in 3 (10%) tests, one of which correlated with true infection. Karius results correlated with other infectious diagnostics (such as viral PCR or cultures) in 10 (33%) cases. Clinically relevant pathogens (not identified by other testing) were identified by Karius in only 3 (10%) cases. There was no statistically significant correlation between pathogen detection by Karius and history of HIV (p=0.27), transplant (p=0.70), malignancy (p= 0.21), or number of days from admission to test (p=0.66)

Table 1

**Figure:**
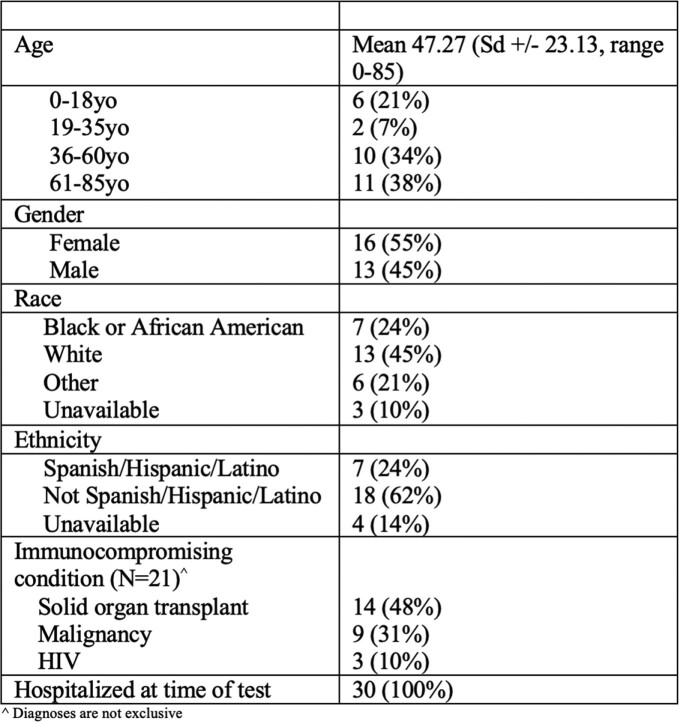

Patient characteristics (N=29 unique patients, 30 tests)

Figure 1

**Figure:**
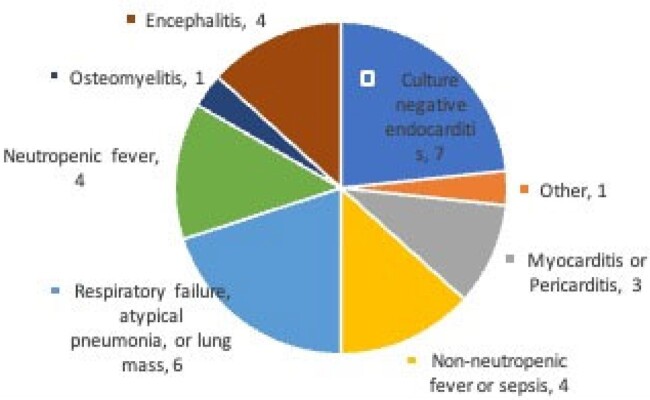

Reasons for Karius test

**Conclusion:**

Metagenomic NGS is an exciting area of diagnostic innovation, but more studies are needed to determine when and where this test is best utilized.

**Disclosures:**

**All Authors**: No reported disclosures

